# Clinical mentorship and TB detection in prison health facilities

**DOI:** 10.5588/ijtldopen.26.0139

**Published:** 2026-08-11

**Authors:** J. Fielder, J. Wanza, T. Muinde, E. Kimutai, M. Muthoni, E. Amdany, C. Ondieki, E. Mungathia, D. Onyango, E. Osoro

**Affiliations:** 1 African Mission Healthcare, Nairobi, Kenya;; 2 Kenya Prisons Service, Nairobi, Kenya;; 3 AIC Kijabe Hospital, Kijabe, Kenya;; 4 LVCT Health, Nairobi, Kenya;; 5 Kisumu County Department of Health, Kisumu, Kenya;; 6 Washington State University, Pullman, WA, USA;; 7 Washington State University Global Health Kenya, Nairobi, Kenya.

**Keywords:** tuberculosis, Kenya, diagnostic practices, diagnostic yield, implementation science

## Abstract

**BACKGROUND:**

TB incidence in prison populations exceeds surrounding communities by factors of up to 100. Early case detection in correctional systems is constrained by gaps in health care worker training and inconsistent application of diagnostic algorithms. Structured clinical mentorship may strengthen diagnostic performance, although evidence from Kenyan correctional settings remains limited.

**METHODS:**

We conducted a retrospective evaluation across 20 prison health facilities in Kenya, comparing TB indicators during 4-month pre- and post-intervention periods following a 3-month clinical mentorship programme. Facility-level outcomes included TB screening coverage, testing coverage, diagnostic modality use, and TB detection among presumptive cases. Analyses were stratified by baseline facility TB detection capacity (≥1 vs. 0 confirmed cases pre-intervention).

**RESULTS:**

Among inmates, TB diagnostic yield was higher in the post-intervention period than in the pre-intervention period (2.6% vs. 5.4%; risk ratio [RR] 2.05, 95% confidence interval: 1.33–3.17), and screening coverage also increased (58.5% vs. 70.3%; RR 1.20; *P* < 0.001). Diagnostic practices shifted toward WHO-recommended modalities. These changes were concentrated in facilities with baseline detection capacity.

**CONCLUSION:**

Clinical mentorship was associated with higher TB diagnostic yield, greater screening coverage, and increased use of WHO-recommended diagnostics during the post-intervention period. Gains were concentrated in facilities with baseline detection capacity.

TB incidence in prison populations exceeds surrounding communities by 10 to 100 times, driven by overcrowding, HIV co-infection, poor ventilation, and delayed health-seeking.^1,2^ Transmission extends beyond incarcerated populations: released individuals and prison staff serve as epidemiologic bridges linking intra-prison dynamics to community spread, complicating national TB control strategies.^2,3^ Effective TB control in correctional settings requires efficient case-finding pathways supported by reliable screening systems and consistent diagnostic capacity. In sub-Saharan African prisons, operational gaps limit uptake of WHO-recommended diagnostics including GeneXpert MTB/RIF and chest radiography.^4,5^ Initial testing with sputum microscopy is not preferred in high-risk populations given its inability to detect rifampicin resistance. Health care workers frequently lack structured training in screening protocols, diagnostic interpretation, and integration of molecular and radiographic findings into case management decisions, contributing to diagnostic delays and missed cases.^5,6^ The Kenya Prisons Service has prioritised TB control, yet empirical evaluation of workforce-focused implementation strategies in Kenyan prison facilities remains limited. GeneXpert MTB/RIF offers rapid, high-sensitivity diagnosis and rifampicin resistance detection,^6^ while chest radiography provides complementary diagnostic information when interpreted by trained personnel. Diagnostic accuracy depends on consistent application of protocols and clinician confidence in interpreting results – skills that require structured, practice-based reinforcement beyond classroom instruction alone.

Clinical mentorship represents a practical approach to strengthening diagnostic performance in resource-limited settings. A systematic review of TB health care provider training evaluations found that most studies assessed only knowledge acquisition, with few examining downstream clinical outcomes.^7^ Unlike stand-alone training, mentorship combines knowledge transfer with hands-on supervision and facility-level coaching, enabling health care workers to apply and refine diagnostic judgment within real-world constraints. Evidence from Uganda and Zambia indicates that mentorship can improve screening adherence, increase use of molecular diagnostics, and raise case detection in correctional settings.^4,5^ Systematic reviews similarly report that mentoring programmes integrating classroom instruction with ongoing supervisory support produce measurable improvements in health care worker performance, particularly in facilities with some baseline capacity.^8^ Evidence from Kenyan correctional facilities, however, remains sparse, and the extent to which structured mentorship is associated with improved diagnostic practices in Kenyan prison health services has not been well described.

We assessed whether implementation of a structured clinical mentorship programme was associated with changes in TB screening coverage, diagnostic testing practices, and TB diagnostic yield across 20 Kenya Prisons Service health facilities. We further examined whether facility baseline TB detection capacity modified patterns of mentorship-associated changes, given known variability in infrastructure readiness across Kenya’s prison health system.

## METHODS

This retrospective pre–post intervention study evaluated associations between clinical mentorship implementation and TB outcomes across 20 prison health facilities in Kenya. The design compared TB control indicators during a 4-month pre-intervention period and a 4-month post-intervention period, with the intervention month excluded from analysis to minimise transition bias. This window captures proximal intervention effects while limiting confounding from broader health system shifts such as policy updates or resource reallocations. Facilities were distributed across six Kenyan administrative regions – Central, Coast, Eastern, Nairobi, Nyanza, and Rift Valley – ensuring representation of regional variation in health care infrastructure, TB burden, and resource availability. All participating facilities provided health care to incarcerated individuals (the primary analysis population), prison staff, and surrounding community members, with TB-related encounters systematically documented in standardised facility registers.

### Study population and sampling

Twenty facilities were selected across six administrative regions (three to four per region), all offering comprehensive TB and HIV services and maintaining standardised TB management registers throughout both observation periods. Registers included the Prison Form-10 (PF10, inmate admission-level TB symptom screening), the Presumptive TB Register (clinical case identification and diagnostic testing records), and the TB4 Register (national treatment initiation documentation). Analyses were stratified by population given fundamental differences in health care access, TB epidemiology, and facility engagement between incarcerated and non-incarcerated individuals. The inmate population comprised all individuals admitted during each observation period and served as the primary analytical focus given consistent facility engagement and stable denominators. Non-inmate populations, prison staff, and community members documented in facility registers demonstrated less consistent health care–seeking patterns, smaller and more variable denominators, and less stable facility engagement; these findings were treated as secondary and interpreted descriptively.

### Data management and outcome definitions

Data were abstracted from the three standardised registers by trained research assistants, with quality checks including cross-verification against source registers and supervisory review. All entries were de-identified before analysis. Three outcomes were evaluated at the facility level for each observation period. TB screening coverage was defined as the proportion of inmate admissions screened for TB symptoms at intake via PF10. TB testing coverage captured the proportion of all presumptive TB cases receiving at least one WHO-recommended diagnostic test (GeneXpert MTB/RIF, chest radiography, or sputum microscopy). The primary outcome, the TB diagnostic yield, represented the proportion of presumptive TB cases registered in the TB4 Register as a new TB diagnosis, including both bacteriologically confirmed and clinically diagnosed cases, consistent with the Global Tuberculosis Dictionary.^9^ This measure reflects diagnostic yield among presumptive cases rather than TB incidence or prevalence. For TB4-based analyses, 12 of 20 facilities had complete data across all observation periods; the eight facilities with incomplete TB4 reporting were excluded using a complete-case approach. Facilities were stratified by baseline detection capacity, defined as whether at least one new TB diagnosis among presumptive inmate cases was recorded in the TB4 register during the pre-intervention period (≥1 diagnosis; n = 12), and facilities with no recorded baseline detection (0 diagnoses; n = 8).

### Statistical analysis

The facility was the unit of analysis. All 20 facilities contributed to analyses of TB screening and testing coverage. Twelve facilities with complete TB4 data across all observation periods contributed to TB diagnostic-yield analyses. Individual-level entries were aggregated to facility-level counts and proportions, with exact binomial 95% confidence intervals (CIs) generated for all outcomes. Pre–post comparisons used Fisher’s exact test, appropriate for sparse cell counts and small denominators. Risk ratios (RRs) with exact 95% CIs were derived from Fisher exact estimates; statistical significance was defined as α = 0.05 for two-sided tests. Heterogeneity in pre–post patterns by baseline detection capacity was explored through stratified analyses, with stratum-specific RRs interpreted descriptively. These subgroup analyses were exploratory and were not intended as confirmatory tests of interaction given the small number of facilities. Non-inmate estimates were generated using identical procedures but interpreted descriptively. All analyses were conducted in R (version 4.0.3).

### Clinical mentorship programme

The clinical mentorship intervention, ‘Tuberculosis in the Era of HIV’, was implemented from April to June 2021 at AIC Kijabe and Maua Methodist hospitals under oversight of the Kenya National Tuberculosis, Leprosy, and Lung Disease Program. A 4-day hospital-based component combined case-based discussions, supervised clinical rotations, and structured review of national TB guidelines, with particular emphasis on chest radiograph interpretation, GeneXpert MTB/RIF utilisation in place of smear microscopy, and management of TB/HIV co-infection. Participants engaged directly in the TB care continuum while receiving real-time feedback from senior clinicians and national TB programme officers. A subsequent 3-month facility-level mentorship phase delivered scheduled virtual and telephone consultations to address operational barriers, refine diagnostic workflows, and monitor facility action plans targeting screening coverage, testing uptake, and case detection.

### Ethical statement

Ethical approval was provided by the Kijabe Hospital Institutional Scientific and Ethical Review Committee (KH/ISERC/02/718/0033/2022), with administrative authorisation from the Kenya Prisons Service and research licensure from the National Commission for Science, Technology, and Innovation (NACOSTI/P/23/23705). As the study involved only retrospective review of existing clinical records, the institutional review board granted a waiver of informed consent. All data were de-identified before analysis and stored securely with restricted access.

## RESULTS

During the pre-intervention period, 23,160 inmate admissions were recorded and 1,238 presumptive TB cases identified, including 168 non-inmates. In the post-intervention period, 25,635 inmate admissions were documented with 1,654 presumptive cases, including 407 non-inmates. Both the Presumptive TB Register and TB4 Register contained entries for inmates and non-inmates, whereas PF10 screening data applied solely to inmates.

### Demographic and clinical characteristics

Among 2,317 inmates identified as presumptive TB cases, baseline characteristics remained consistent across periods. Men comprised 97.0% of the pre-intervention cohort and 98.3% post-intervention. Mean age was 36.9 years pre-intervention and 37.6 years post-intervention (*P* = 0.742). HIV testing uptake was documented in 67.7% of inmates pre-intervention and 64.0% post-intervention. Additional socio-demographic characteristics are presented in Supplementary Data Table 2.

### TB diagnostic yield

**Figure 1.**
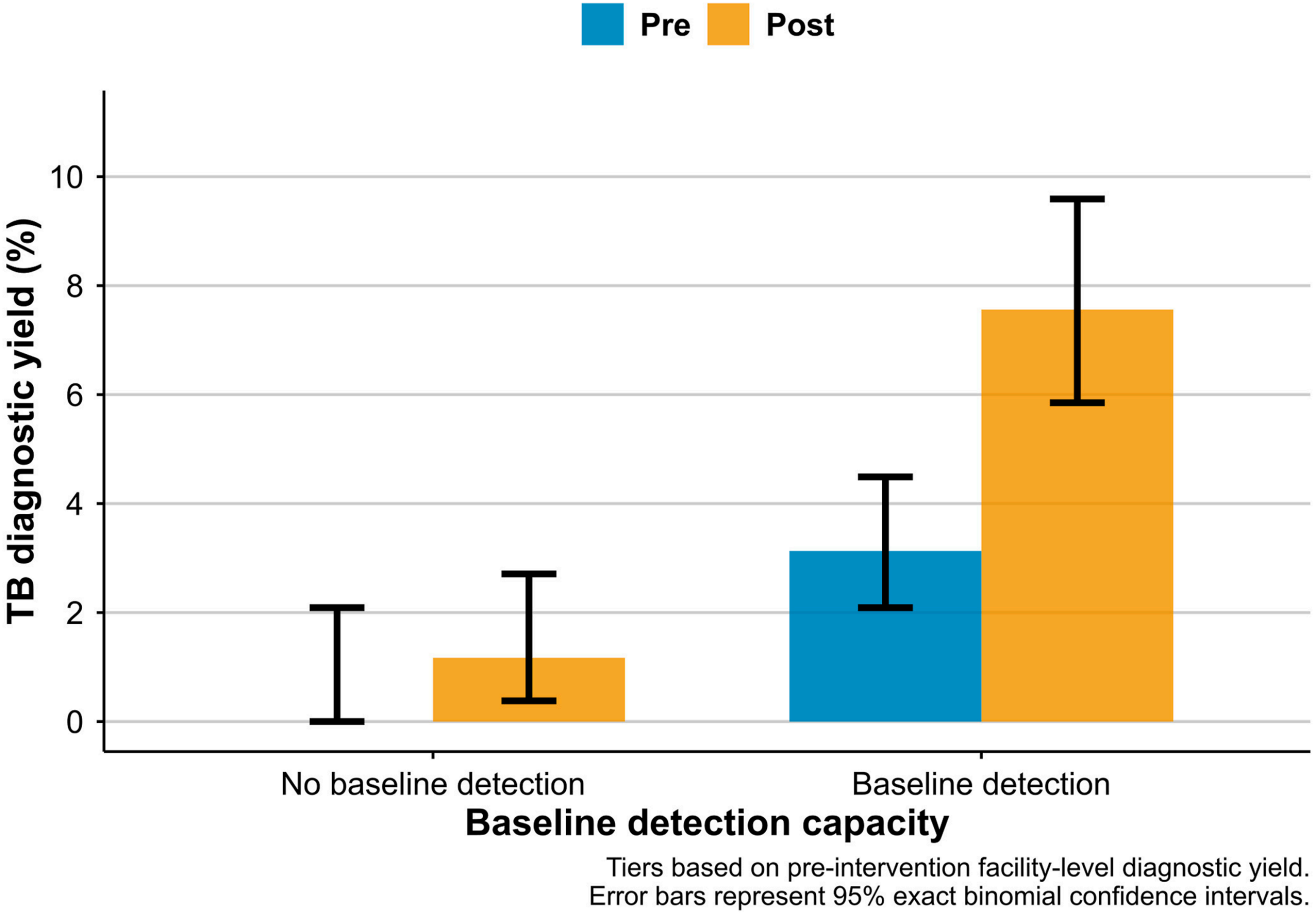
Facility-level TB diagnostic yield among presumptive inmate cases, stratified by baseline detection capacity. Facilities with at least one TB diagnosis recorded during the pre-intervention period (n = 12) increased from 3.1% to 7.6% (RR 2.41, *P* < 0.001). Facilities with no recorded TB diagnosis during the pre-intervention period (n = 8) changed from 0% to 1.2% (*P* = 0.328).

Among inmates, the proportion of presumptive TB cases diagnosed with TB was 2.6% (95% CI: 1.7%–3.8%) pre-intervention and 5.4% (95% CI: 4.2%–6.8%) post-intervention (RR 2.05; 95% CI: 1.33–3.17; *P* = 0.001), indicating more than doubling of the diagnostic yield concurrent with clinical mentorship implementation (Table). Detection gains differed markedly by baseline detection capacity. In Active-Detection Baseline facilities (n = 12), detection rose from 3.1% (95% CI: 2.1%–4.5%) to 7.6% (95% CI: 5.9%–9.6%) (RR 2.41; *P* < 0.001). In contrast, Zero-Detection Baseline facilities (n = 8) showed only a small increase from 0% to 1.2% (95% CI: 0.4%–2.7%; *P* = 0.328). Of the 39 additional confirmed cases identified post-intervention, 34 (87%) occurred in facilities with established baseline detection capacity, which accounted for 68% of presumptive cases (Figure 1, Supplementary Data Table S2).

**Table.** TB control outcomes among inmates before and after clinical mentorship across 20 Kenyan prison health facilities. Percentages are based on exact binomial 95% confidence intervals (CI). TB screening coverage is proportion of admitted inmates screened at entry (Prison Form-10 register). TB testing coverage is proportion of presumptive TB cases receiving at least one diagnostic test (GeneXpert MTB/RIF, chest radiography, or sputum microscopy). TB diagnostic yield is proportion of presumptive TB cases documented in the TB4 register as newly diagnosed TB and initiating treatment. Diagnosed TB includes both bacteriologically confirmed and clinically diagnosed cases.

Outcome	Measure	Pre-intervention	Post-intervention	Risk ratio (95% CI)	*P* value^A^
TB screening coverage	% of admissions screened (95% CI)	58.5% (57.8–59.1)	70.3% (69.8–70.9)	1.20 (1.19–1.22)	<0.001
	n screened/n admitted	13,544/23,160	18,028/25,635		
TB testing coverage	% of presumptive cases tested (95% CI)	80.7% (78.2–83.0)	83.1% (80.9–85.1)	1.03 (0.99–1.07)	0.191
	n tested/n presumptive	863/1,070	1,036/1,247		
TB diagnostic yield	% of presumptive cases confirmed (95% CI)	2.6% (1.7–3.8)	5.4% (4.2–6.8)	2.05 (1.33–3.17)	0.001
	n confirmed/n presumptive	28/1,070	67/1,247		

A Fisher’s exact test for pre- versus post-intervention comparison at facility level.

Among non-inmates, the diagnostic yield declined from 46.2% (78 of 169 cases) to 30.7% (126 of 410 cases) post-intervention (RR 0.67; 95% CI: 0.54–0.82; *P* < 0.001). This decline occurred concurrent with a 143% expansion in the presumptive case denominator, with only a 62% increase in confirmed cases.

### TB testing practices and diagnostic modality use

**Figure 2.**
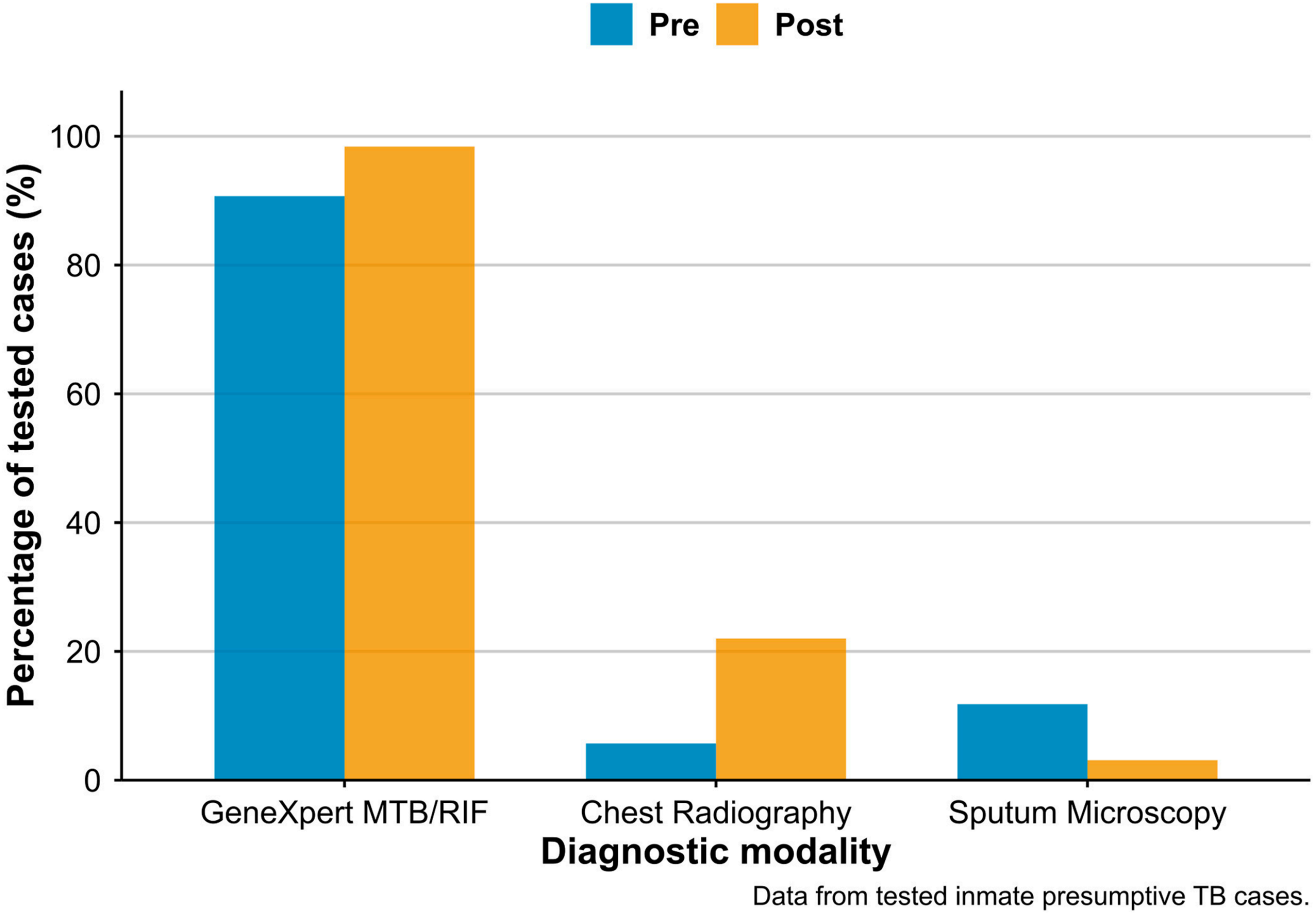
Diagnostic modality use among tested inmate presumptive TB cases in pre- and post-intervention periods. GeneXpert MTB/RIF: 90.7%–98.4%. Chest radiography: 5.7%–22.0%. Sputum microscopy: 11.8%–3.1%. Data from inmate presumptive TB cases. Percentages calculated among tested cases.

Among inmates, the proportion receiving at least one diagnostic test increased from 80.7% (95% CI: 78.2%–83.0%) to 83.1% (95% CI: 80.9%–85.1%) (RR 1.03; 95% CI: 0.99–1.07; *P* = 0.191), indicating stable and already-elevated testing coverage across both periods (Table). Diagnostic modality use shifted among tested presumptive cases (Figure 2). GeneXpert MTB/RIF use increased from 90.7% (95% CI: 88.6%–92.6%) to 98.4% (95% CI: 97.4%–99.1%). Chest radiography expanded from 5.7% (95% CI: 4.5%–7.1%) to 22.0% (95% CI: 19.9%–24.2%), a nearly fourfold increase. Sputum microscopy declined from 11.8% (95% CI: 10.0%–13.8%) to 3.1% (95% CI: 2.3%–4.1%).

### TB screening coverage

TB symptom screening coverage at inmate admission increased from 58.5% (95% CI: 57.8%–59.1%) to 70.3% (95% CI: 69.8%–70.9%) (RR 1.20; 95% CI: 1.19–1.22; *P* < 0.001), representing an absolute increase of 4,484 individuals screened (Table).

## DISCUSSION

Following clinical mentorship across 20 Kenya Prisons Service health facilities, TB diagnostic yield doubled in the post-intervention period, accompanied by a 20% expansion in TB symptom screening coverage and a broader use of WHO-recommended diagnostic practices compared to the pre-intervention period. These improvements occurred alongside stable and already-elevated testing coverage, suggesting that the intervention primarily influenced diagnostic quality and case confirmation rather than initial testing uptake.

Changes in diagnostic practices were evident across the case-finding pathway in the post-intervention period. A particularly striking finding was the substantial reorientation of modality use among tested cases: chest radiography expanded nearly fourfold, GeneXpert MTB/RIF approached universality, and sputum microscopy declined substantially. These shifts are consistent with the mentorship curriculum’s emphasis on radiograph interpretation and GeneXpert result use in routine care. Recent prison-based data from Tajikistan also showed higher screening yield among symptomatic individuals and greater yield when chest X-ray was used, which is consistent with the expanded radiography observed here.^10^ Together, these findings suggest that the mentorship package may have influenced how available diagnostic tools were used within routine prison TB evaluation.

Stratified analyses found that gains were concentrated in facilities with pre-existing detection capacity. Facilities with at least one TB diagnosis recorded during the pre-intervention period showed a 2.4-fold increase in diagnostic yield, whereas facilities with no recorded diagnoses showed little change despite receiving the same mentorship package. This pattern suggests that training was more readily translated into practice where some diagnostic capacity was already in place. A recent prison-based study from Brazil likewise points to the importance of broader system conditions, including staffing, confirmatory testing, transfer-related interruptions, and treatment supervision.^10,11^

Non-inmate patterns differed substantially from those among inmates and highlight an important methodological consideration for prison health research. Although absolute confirmed cases increased by 62%, detection rates declined from 46.2% to 30.7% (RR 0.67; *P* < 0.001), reflecting a 143% expansion in the presumptive case denominator as non-inmate presentations to prison facilities nearly tripled. Demographic shifts likely contributed, with individuals under 18 years growing from 9.1% to 21.9% (*P* < 0.001) – a group with inherently lower TB positivity. Non-inmate engagement with prison health services is episodic and heterogeneous, producing unstable denominators compared with the consistently enrolled inmate population. Facility-level detection metrics must therefore be interpreted in relation to population-specific denominators and demographic composition. Non-inmate findings are secondary and descriptive, and the inmate population remains the more appropriate basis for assessing mentorship-associated effects.

These infrastructure-dependent findings extend evidence from Uganda, where mentorship-based training enhanced TB case detection^4^ and one-on-one mentorship improved facility-level performance indicators in a cluster-randomised trial,^12^ and from Zambia, where systematic screening with diagnostic capacity building improved detection in prison settings.^5^ Systematic reviews similarly demonstrate that mentorship integrating classroom instruction with structured on-site support enhances health care worker performance, particularly when supported by adequate facility infrastructure.^8^ A cluster-randomised trial in South Africa found that experiential staff training did not improve TB outcomes, potentially reflecting health system barriers not addressed by training alone,^13^ while laboratory capacity building in Tanzania improved case detection when implemented alongside collaborative health system strengthening.^14^ Collectively, this evidence indicates that pre-existing organisational readiness and supervisory capacity are necessary conditions for translating individual training into sustained facility-level practice change.

Limitations include voluntary facility enrolment, which may have favoured higher-capacity sites, the absence of concurrent controls, and short 4-month pre- and post-intervention observation periods. The post-intervention period was too short to determine whether the observed changes were maintained over time. The analytic design could not disaggregate the relative contributions of the 4-day classroom component and the 3-month facility-based mentorship phase. Both bacteriologically confirmed and clinically diagnosed TB cases were included, consistent with WHO algorithms for resource-limited settings, although clinical diagnosis carries some risk of overdiagnosis. In addition, restricting TB4-based analyses to facilities with complete reporting may have biased estimates toward sites with stronger documentation systems and may limit generalisability to lower-performing or less consistently reporting facilities. We did not systematically document changes in GeneXpert availability, cartridge supply, radiography access, or equipment functionality during the study period, so some of the observed diagnostic changes may reflect concurrent resource shifts rather than mentorship alone. Although the mentorship programme was implemented in 2021, the issues examined here remain relevant. Recent prison-based studies continue to highlight the importance of screening pathways, diagnostic yield, and continuity of care in correctional TB control.^10,11^ The current service delivery approaches and infrastructure remain largely as they were in 2021. TrueNat molecular testing has been introduced within select prison facilities, but not at those included in this report. AI-assisted radiographic interpretation is available intermittently.

## CONCLUSION

Our findings suggest that structured mentorship was associated with short-term improvements in TB diagnostic practices and diagnostic yield among presumptive inmate cases, particularly in facilities with baseline detection capacity. Mentorship is likely to be more effective when supported by complementary facility-level readiness, including reliable diagnostic access, functional radiography, and ongoing supervisory systems.

## Supplementary Material
