# Supplementary material for: Clinical mentorship and TB detection in prison health facilities
Source: IJTLD Open. 2026 Aug 11;3(8):520–5. doi: 10.5588/ijtldopen.26.0139 (PMC13460427; doi:10.5588/ijtldopen.26.0139)
Supplement: Supplementary file 1 [file ijtldopen26-0139_supplementarydata1.pdf]

SUPPLEMENTARY DATA

Table S1: Sociodemographic and clinical characteristics of inmate and non-inmate presumptive TB cases by intervention period

	Inmates			Non-inmates		
**Variable**	**Pre-intervention (n=1070)**	**Post-intervention (n=1247)**	**p value**	**Pre-intervention (n=168)**	**Post-intervention (n=407)**	**p value**
**Sex**			0.068			0.438
Female	32 (3.0%)	21 (1.7%)		78 (46.4%)	210 (51.6%)	
Male	1038 (97.0%)	1226 (98.3%)		89 (52.9%)	196 (48.2%)	
Missing	–	–		1	1	
**Age, years (mean, 95% CI)**	36.9 (36–38)	37.6 (37–38)	0.742	32.8 (33–37)	31.4 (31–34)	0.356
Missing	–	–		3	14	
**Age group**			0.892			<0.001
<18 years	12 (1.1%)	11 (0.9%)		15 (9.1%)	89 (21.9%)	
18–30 years	368 (34.4%)	413 (33.1%)		49 (29.7%)	87 (21.4%)	
31–40 years	336 (31.4%)	396 (31.7%)		54 (32.7%)	96 (23.6%)	
41–50 years	218 (20.4%)	217 (17.4%)		26 (15.8%)	92 (22.6%)	
>50 years	136 (12.7%)	210 (16.8%)		23 (13.9%)	42 (10.3%)	
Missing	–	–		3	14	
**HIV test status**			0.089			0.084
Tested	725 (67.7%)	798 (64.0%)		131 (78.2%)	357 (87.5%)	
Not tested	297 (27.8%)	405 (32.5%)		24 (14.1%)	26 (6.4%)	
Known positive	35 (3.3%)	25 (2.0%)		13 (7.7%)	23 (5.6%)	
Declined	12 (1.1%)	19 (1.5%)		0 (0.0%)	1 (0.5%)	
Missing	–	–		12	15	

Data are n (%) unless otherwise indicated. Percentages calculated among non-missing observations.
TB=tuberculosis. CI=confidence interval.

Table S2: TB diagnostic yield stratified by baseline facility detection capacity

**Baseline detection capacity**	**n facilities**	**Pre-intervention**	**Post-intervention**	**Risk ratio (95% CI)**	p value[1]
**Baseline detection**					
Diagnostic yield, % (95% CI)	12	3.1% (2.1–4.5)	7.6% (5.9–9.6)	2.41 (1.53–3.80)	<0.001
Diagnosed /presumptive	–	28/895	62/820		
**No Baseline detection**					
Diagnostic yield, % (95% CI)	8	0% (0–2.1)	1.2% (0.4–2.7)	–	0.328
Diagnosed /presumptive	–	0/175	5/427		

[1] Fisher's exact test.
[2] Baseline detection: ≥1 TB diagnosis recorded pre-intervention. No baseline detection: 0 TB diagnoses recorded pre-intervention.
95% CIs are exact binomial. Risk ratio was not estimated for Zero-Detection Baseline facilities because the pre-intervention rate was zero, making the ratio undefined. TB=tuberculosis. CI=confidence interval.
